# Prediction of Sarcopenia Using Multiple Biomarkers of Neuromuscular Junction Degeneration in Chronic Obstructive Pulmonary Disease

**DOI:** 10.3390/jpm11090919

**Published:** 2021-09-15

**Authors:** Asima Karim, Tahir Muhammad, Rizwan Qaisar

**Affiliations:** 1 Basic Medical Sciences, College of Medicine, University of Sharjah, Sharjah 27272, United Arab Emirates; akarim@sharjah.ac.ae; 2 Department of Physiology and Cell Biology, University of Health Sciences, Lahore 53720, Pakistan; 3 Department of Biochemistry, Gomal Medical College, Gomal University, Dera Ismail Khan 30130, Pakistan; drtahir82@gmail.com

**Keywords:** BDNF, CAF22, chronic obstructive pulmonary disease, GDNF, sarcopenia, skeletal muscle

## Abstract

Patients with chronic obstructive pulmonary disease (COPD) present with an advanced form of age-related muscle loss or sarcopenia. Among multiple pathomechanisms of sarcopenia, neuromuscular junction (NMJ) degradation may be of primary relevance. We evaluated the circulating biomarkers of NMJ degradation, including c-terminal agrin fragment -22 (CAF22), brain-derived neurotrophic factor (BDNF), and glial cell line-derived neurotrophic factor (GDNF) as predictors of sarcopenia in COPD during pulmonary rehabilitation (PR). Male, 61–77-year-old healthy controls and patients of COPD (*n* = 77–84/group) were recruited for measurements of circulating CAF22, BDNF, and GDNF levels. Functional assessment and measurements of plasma biomarkers were performed at diagnosis and following six months of PR. CAF22 levels were elevated while BDNF and GDNF levels were reduced in COPD patients at diagnosis, which were incompletely restored to normal levels following PR. These biomarkers showed varying degrees of associations with indexes of sarcopenia and functional recovery during PR. Logistic regression revealed that the combined use of three biomarkers enhanced the diagnostic accuracy of sarcopenia better than single biomarkers. Altogether, measurements of plasma CAF22, BDNF, and GDNF may be helpful for the accurate diagnosis of sarcopenia and functional capacity in COPD during PR.

## 1. Introduction

Skeletal muscle dysfunction is a common systemic manifestation in patients with chronic obstructive pulmonary disease (COPD) [1]. Loss of muscle mass and strength in these patients negatively affects the exercise capacity and functional independence, leading to diminished quality of life. Both the ventilatory and peripheral muscles are affected, resulting in increased hospitalization and mortality, independent of airway obstruction. The loss of muscle mass is more severe in peripheral muscles, and causes devastating effects on the quality of life [2]. Muscle quality mirrors generalized health in COPD and is thus associated with conditions such as exacerbation, hospitalization, and decline of other organ systems [3].

Muscle weakness and atrophy are the primary components of age-related muscle loss of sarcopenia [4]. However, comorbidities such as COPD can accelerate the onset and/or severity of sarcopenia in the elderly [5]. The European working group on sarcopenia in older people (EWGSOP2) recommends distinguishing secondary sarcopenia due to comorbidities from primary sarcopenia due to ageing [6]. Although the onset of COPD is in the middle to late age, the accelerated sarcopenia phenotype in COPD is independent of age [7]. Additionally, a crosstalk between COPD and sarcopenia is suggested so that the presence of sarcopenia may exacerbate the severity of COPD and reduce the efficacy of rehabilitation programs. These findings necessitate the early evaluation of sarcopenia to improve therapeutic efficacy and functional independence in COPD.

Given the labor-intensive diagnostic procedure of sarcopenia in clinical settings, short, objective, and easily administrable tests might be helpful in the early assessment of sarcopenia. The SARC-F questionnaire and short physical performance battery (SPPB) score are valuable measures of muscle strength and physical capacity. SARC-F is a self-reporting questionnaire based on symptom scores and features associated with sarcopenia [8]. SPPB is a composite test used for the assessment of functional capacity in daily life [6]. Both SARC-F and SPPB are objective measures of muscle strength and physical capacity, and are recommended by the EWGSOP2 for evaluation of sarcopenia [6].

The disintegration of the neuromuscular junction (NMJ) is a central focal point in the multifactorial etiology of sarcopenia [9]. We have previously shown that NMJ disintegration can trigger muscle atrophy and weakness in ageing, and conditions associated with increased oxidative stress [10]. Notably, the muscle shows reduced oxidative capacity, fiber-type grouping, and signs of denervation. Several lines of evidence suggest that the NMJ instability is an essential contributor to sarcopenia associated with COPD. First, the skeletal muscle in COPD patients shows the fiber-type grouping similar to recurring denervation-reinnervation cycles driven by NMJ disruption in ageing and other pathologies [11]. Second, tobacco smoking, a leading cause of COPD, is shown to initiate NMJ degeneration in human and animal models [11]. Third, an upregulation of MUSK, an NMJ-related gene, has been reported in muscle tissues in COPD, consistent with functional denervation [12]. Fourth, an elevation of plasma c-terminal agrin fragment-22 (CAF22), a biomarker of NMJ disintegration, is reported in COPD patients [13]. Together, these findings highlight the pivotal role of NMJ degradation in sarcopenia associated with COPD and indicate that monitoring NMJ integrity can be a valuable tool to assess sarcopenia in COPD. Compared to muscle biopsies, plasma biomarkers offer an easy and objective assessment of NMJ and muscle integrity in clinical settings. Further, owing to the multifactorial etiology of NMJ degradation, more than one biomarker is required to accurately evaluate NMJ and muscle health in COPD.

Brain-derived neurotrophic factor (BDNF) and glial cell line-derived neurotrophic factor (GDNF) are released by the motor neuron, muscle fibers, and peri-synaptic Schwann cells (PSCs) and modulate neural plasticity by supporting maturation and innervation at the NMJ. Upon local release at the NMJ, these neurotrophic factors protect against denervation and muscle loss in catabolic conditions [14]. This protective effect is lost in sarcopenia as circulating BDNF, and GDNF levels decrease in advancing age in both genders [14]. Thus, the reduced upregulation of BDNF and GDNF in the elderly can potentially contribute to sarcopenia in COPD.

CAF22 has recently emerged as a potential circulating biomarker of NMJ degradation and muscle loss. CAF22 is a byproduct of agrin, a neuronal proteoglycan involved in the precise alignment of the motor endplate and acetylcholine receptors [13]. During NMJ remodeling, agrin is cleaved by a neuronal protease, neurotrypsin, into two sub-fragments. CAF22 is the smaller of the two sub-fragments and has a close association between circulating CAF22 levels and many functional and clinical indexes of physical capacity [13,14,15]. We have recently shown a negative correlation of plasma CAF22 level with sarcopenia phenotype in respiratory diseases, including COPD [1,13]. Specifically, higher plasma CAF22 levels are associated with reduced muscle mass, strength, and gait speed.

Since the NMJ plasticity is regulated by more than one cell type, including motor neurons, PSCs, and muscle fibers, multiple NMJ biomarkers are required to evaluate sarcopenia in COPD accurately. However, to our knowledge, no study has investigated the predictive potential of multiple NMJ biomarkers in diagnosing sarcopenia and physical capacity. The current study aims to address this gap by evaluating circulating BDNF, GDNF, and CAF22 levels as potential biomarkers of sarcopenia in COPD. We took a dynamic approach and investigated the NMJ biomarkers during disease course in COPD patients undergoing pulmonary rehabilitation (PR) and pharmacotherapy. We hypothesized that plasma NMJ biomarkers may help evaluate sarcopenia and functional capacity in COPD during PR. Specifically, we evaluated the plasma BDNF, GDNF, and CAF22 levels with changes in muscle mass, strength, and walking speed in COPD during PR. We also used logistic regression to group these biomarkers into a panel to enhance the diagnostic accuracy of sarcopenia. Additionally, we examined the diagnostic values of SARC-F and SPPB in clinical sarcopenia.

## 2. Materials and Methods

### 2.1. Study Design and Participants

**Figure 1:**
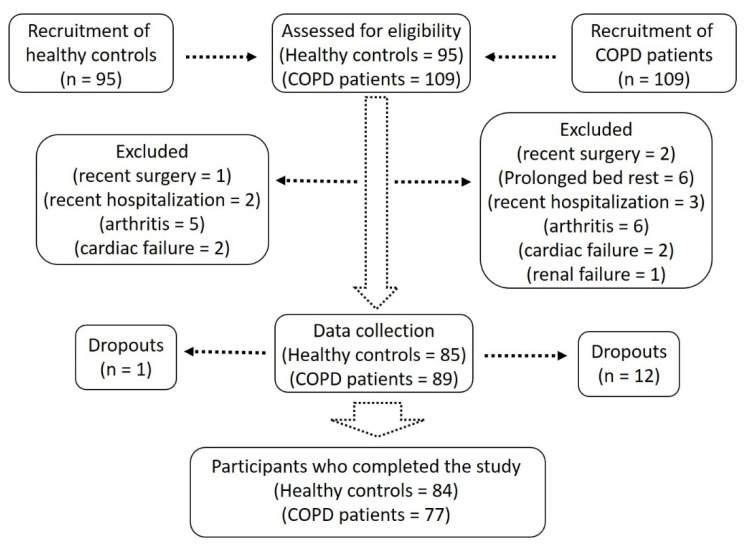
Flow diagram of the study participants.

We recruited male, 61–77-year-old healthy controls (*n* = 84) and patients with COPD (*n* = 77) at the Gomal Medical College, Dera Ismail Khan, after approval by the regional ethical committee. Data was collected from structural interviews, clinical examinations, laboratory investigations, and measurements of physical parameters. Healthy controls were matched for age and BMI and selected from a cohort described previously [1]. COPD was defined as FEV_1_%/forced vital capacity (FVC) < 0.7 with persistent respiratory symptoms according to the GOLD guidelines [16]. Patients with COPD were investigated at the time of diagnosis and six months later during the PR phase, while the healthy controls were only investigated once during the study. The PR was a composite program and included exercise training, education, and psychosocial support with at least three 2–4 h per week sessions. The exercises included high-intensity continuous training involving walking, a treadmill, or a stationary bicycle for at least 30 min to achieve 60–80% of the peak work rate [17]. Based on the definition by EWGSOP, sarcopenia was defined as low muscle strength (Handgrip strength; HGS < 27 kg), low muscle quantity (Appendicular skeletal mass index; ASMI < 7 kg/m^2^), and low physical performance (SPPB ≤ 8 and/or gait speed ≤ 0.8 m/s) [6,18]. Subjects with stable COPD were included, while those with unstable COPD (infection, exacerbation and/or hospitalization in the past one month), arthritis, renal or cardiac failure, prolonged bed rest, and major surgeries within the past eight weeks were excluded (Figure 1) [19].

Body mass index (BMI) was calculated as kg/m^2^. Appendicular skeletal muscle mass (ASM) and fat mass were calculated with a bioelectrical impedance analysis scale (RENPHO, Dubai, UAE). ASM was divided by height square to get ASMI. SARC-F questionnaire was used as an independent and rapid diagnostic tool for sarcopenia [20]. The questionnaire investigates various physical performance measures, as each measure is given a score from 0 to 2 with a maximal score of 10. A score ≥ 4 was taken as a predictor of sarcopenia. Four participants dropped out during the study due to exacerbation or death and were excluded from the final analysis. Written informed consent was obtained from all study participants. This study was conducted in accordance with the declaration of Helsinki [21].

### 2.2. HGS and Body Composition

HGS was measured by a digital handgrip dynamometer (CAMRY, South El Monte, CA, USA) as described before [1,13]. ASM, fat mass, and ASMI were calculated with the bioelectrical impedance analysis scale (RENPHO, Dubai, UAE) [13].

### 2.3. Spirometry

The FEV_1_ and FVC were measured using a portable spirometer (Contec SP10, Shanghai, China), according to standards set by the American Thoracic Society [22]. The participants were instructed to inhale maximally until the lungs were full, followed by forceful exhalation into the spirometer until no air could be exhaled [23]. This was performed a minimum of three times, and the severity grading was based on FEV_1_% of predicted values according to the GOLD criteria into GOLD 1-4 [24].

### 2.4. Measurement of Physical Performance

The SPPB score assessed physical performance. This battery comprises three timed tests: 4-m walking speed, balance, and chair-stand tests. Timed results from each trial were rescored from zero (worst performers) to four (best performers). The sum of the results from the three categorized tests (ranging from 0 to 12) was used for the present analyses, as described elsewhere [25].

### 2.5. Measurement of Plasma Biomarkers

Plasma samples were analyzed using ELISA kits for CAF22 (NTCAF, ELISA, Neurotune, Schlieren-Zurich, Switzerland), BDNF (Cat # ab212166, Abcam, Abu Dhabi, UAE), and GDNF (Cat # ab100525, Abcam, Abu Dhabi, UAE) according to the manufacturer’s instructions.

### 2.6. Measurements of Plasma 8-Isoprostanes, CRP, and Creatine Kinase

We used ELISA to measure 8-isoprostanes (Cayman Chemical, Ann Arbor, MI, USA) and c-reactive proteins (CRP) (R&D Systems, Minneapolis, MN, USA) levels and biochemical assays to measure creatine kinase (CK) levels, as described previously [5].

### 2.7. Statistical Analysis

Anthropometric measurements of the participants were presented using mean and standard deviation as data met the assumption for normality. Analysis of variance was used to compare groups, and Pearson correlation was employed to determine the strength of the relationship between individual cohorts and various physical and biochemical parameters. The relationship between variables was analyzed by simple and multiple regression analysis. A two-sample *t*-test for percent was used to compare SPPB scores among the groups. Data were analyzed using GraphPad Prism 8, and the *p*-value < 0.05 was statistically significant.

## 3. Results

### 3.1. Characteristics of the Participants

The basic characteristics of the study population are summarized in Table 1. At the time of diagnosis, the patients with COPD presented with lower ASM, ASMI, and the phase angle than the healthy controls (all *p* < 0.05). These patients also had reduced physical parameters, including HGS, walking speed, and daily step count. PR restored the ASM and phase angle in COPD patients without affecting the ASMI. We also report a significant, albeit incomplete recovery of HGS and walking speed with PR, although the measurements were still lower than the healthy controls (Table 1).

**Figure 2:**
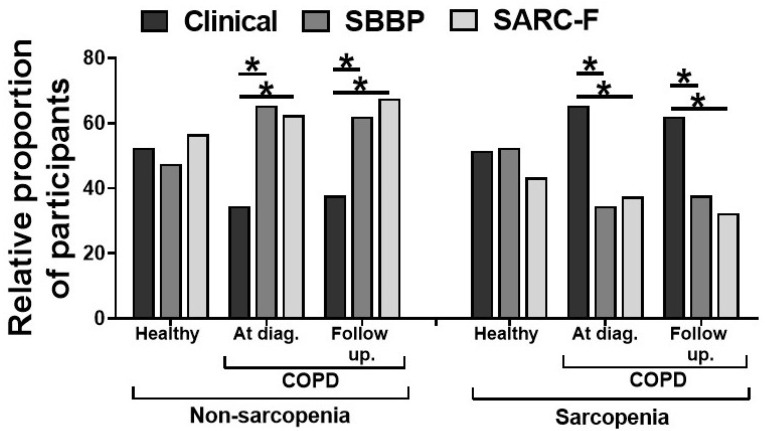
The relative proportion of sarcopenia as defined clinically, by short physical performance battery score (≤8) and the SARC-F score (≥4) in healthy controls and patients with COPD at diagnosis and following pulmonary rehabilitation (*n* = 77–84/group). Values are expressed in percentages, * *p* < 0.05.

The Patients with COPD also showed significantly higher plasma 8-isoprostanes, CRP, and CK levels at the time of diagnosis, which were incompletely restored to baseline levels with PR (Table 1). We next measured the diagnostic efficacy of SPPB (score ≤ 8 was considered as sarcopenia) and SARC-F (score ≥ 4 was considered as sarcopenia) in detecting clinical sarcopenia (EWGSOP criteria). The incidence of sarcopenia measured by the three criteria was similar in the healthy controls (Figure 2). However, patients with COPD had a significantly higher incidence of clinical sarcopenia when compared to SPPB and SARC-F criteria (*p* < 0.05). PR had no significant effects on the comparative incidence of sarcopenia measured by three criteria in COPD patients (Figure 2).

### 3.2. Changes in Circulating Biomarkers Levels in COPD

**Figure 3:**
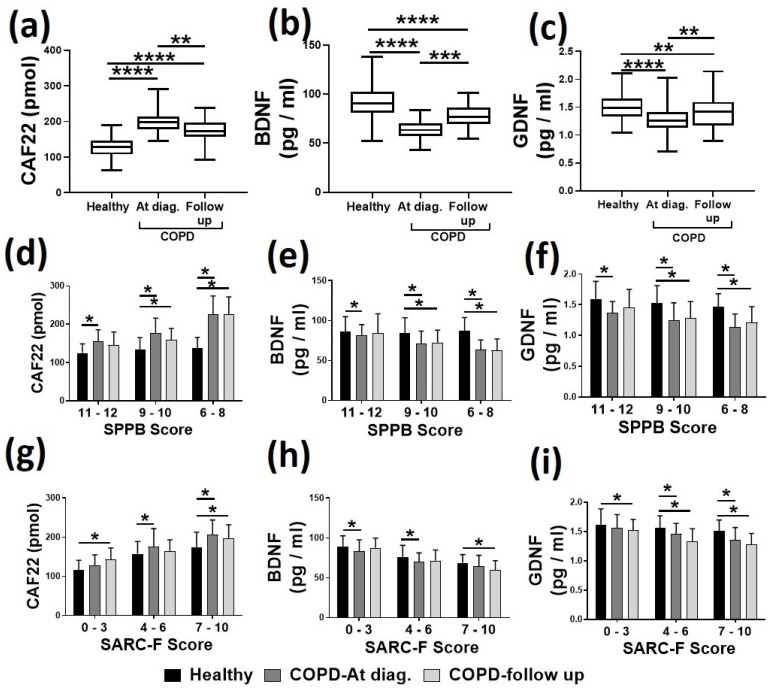
Comparison of circulating CAF22 (**a**,**d**,**g**), BDNF (**b**,**e**,**h**), and GDNF (**c**,**f**,**i**) levels in healthy controls and patients with COPD (*n* = 77–84/group). The biomarkers levels were higher in COPD patients at diagnosis (Dx) and partially restored to normal levels with pulmonary rehabilitating. Poor performance on SBBP (**d**,**e**,**f**) and SARC-F (**g**,**h**,**i**) were associated with higher CAF22 and lower BDNF and GDNF levels. Values are expressed as mean ± SD, one-way analysis of variance. * *p* < 0.05, ** *p* < 0.01, *** *p* < 0.001, **** *p* < 0.0001.

For plasma analysis, we investigated the circulating CAF22, BDNF, and GDNF levels in a total of 231 samples from healthy controls and patients with COPD. The selection of biomarkers was based on published literature and our recent work in patients with respiratory diseases [13,14]. When compared to healthy controls, the patients with COPD had significantly higher levels of CAF22 (57.2% higher, *p* < 0.05) and lower levels of BDNF and GDNF (30.3 and 15.5% lower, respectively, *p* < 0.05) at the time of diagnosis *(*Figure 3a–c).

PR resulted in the incomplete recovery of these biomarkers levels as the differences from the normal levels were reduced for CAF22 (38.4% higher, *p* < 0.05), BDNF, and GDNF (14.7 and 6.3% lower, respectively, *p* < 0.05). We next correlated the levels of biomarkers to the amount of physical activity as measured by SPPB and SARC-F criteria. In general, the COPD subjects with higher SPPB or lower SARC-F scores indicating reduced physical activity had higher CAF22 and lower BDNF and GDNF levels than healthy controls (Figure 3d–g). Thus, PR resulted in a slight restoration of these biomarkers levels for various categories of physical activity.

### 3.3. Evaluation of Sarcopenia Using a Cumulative Risk Score of Three Biomarkers

**Figure 4:**
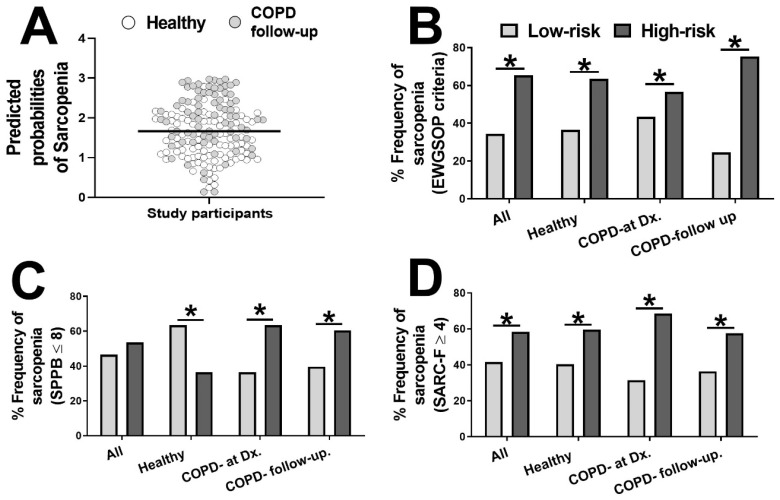
Cumulative risk score for all the participants based on three biomarkers (CAF22, BDNF, and GDNF). The median risk score for the scatter plot of healthy controls and COPD patients at follow-up was 1.583, which was applied to divide the participants into high- and low-risk groups (**A**). The relative proportion of sarcopenia as defined clinically (**B**), SPPB ≤ 8 (**C**), or SARC-F score ≥ 4 (**D**) in the two risk groups in the healthy controls and patients with COPD at diagnosis (Dx) and follow-up (*n* = 77–84/group). * *p* < 0.05.

The combination of three biomarkers into a single score can potentially improve the evaluation of sarcopenia. We applied logistic regression coefficients to the circulating CAF22, BDNF, and GDNF levels to generate predicted probabilities of individual biomarkers for clinical sarcopenia. We next generated the risk scores for sarcopenia by adding the predictive probabilities of individual biomarkers for diagnosis of clinical sarcopenia in all subjects. We obtained a median cut-off value of 1.664 used to divide the participants into high-risk and low-risk groups (Figure 4A).

A significantly higher proportion of COPD patients was found in the higher-risk group. We next measured the relative proportion of clinical sarcopenia in the two risk categories and found a significantly higher incidence of sarcopenic individuals in the high-risk group, independent of disease status (Figure 4B). The proportion of sarcopenic individuals in the high-risk group was still higher when the sarcopenia was defined according to SBBP (Figure 4C) and SARC-F (Figure 4D) criteria, indicating the usefulness of the biomarker panel in evaluating sarcopenia.

### 3.4. Significance of the Biomarker Panel in Diagnosis of Sarcopenia

**Figure 5:**
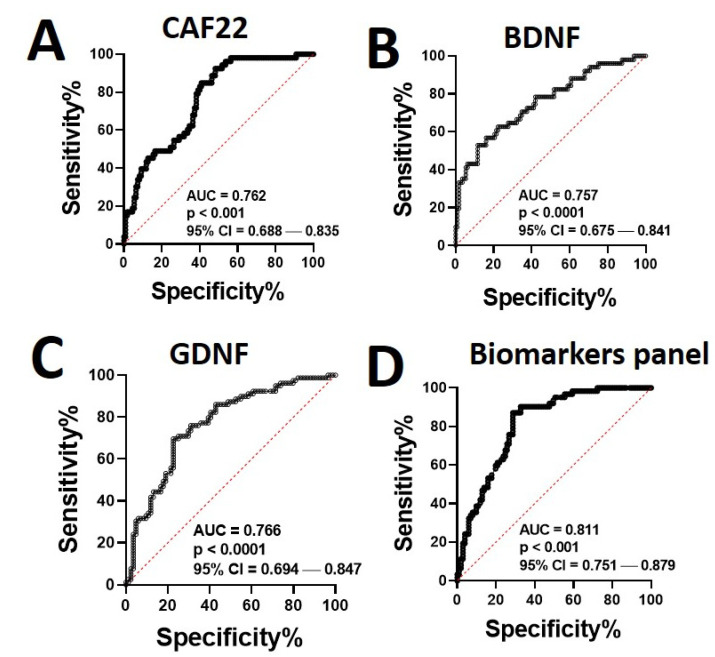
Significance of cumulative risk score for all the participants based on three biomarkers (CAF22, BDNF, and GDNF). Receiver operating characteristic (ROC) curves for CAF22 (**A**), BDNF (**B**), GDNF (**C**), and the biomarkers panel (**D**) in healthy controls and patients with COPD at diagnosis (Dx) and follow-up (*n* = 77–84/group). The area under the curve (AUC) was calculated for each group to determine the significance of the biomarkers panel in the diagnosis of sarcopenia.

We next assessed the potential of the NMJ biomarkers in diagnosing sarcopenia by generating ROC curves using the healthy controls and COPD patients at follow-up (Figure 5).

We obtained significant values for the areas under the ROC curve (AUCs) obtained for CAF22 (AUC = 0.762, *p* < 0.001, CI = 0.688–0.835) (Figure 5A), BDNF (AUC = 0.757, *p* < 0.001, CI = 0.675–0.841) (Figure 5B), and GDNF (AUC = 0.766, *p* < 0.001, CI = 0.694–0.847) (Figure 5C). The AUC was further increased for the biomarker panel (AUC = 0.811, *p* < 0.001, CI = 0.751–0.879) (Figure 5D), indicating that the biomarker panel has an advantage over individual biomarkers in the diagnosis of sarcopenia. To assess the diagnostic potential of the NMJ biomarkers in varying severity of sarcopenia, we next generated the ROC curves for healthy controls and COPD patients during PR (Table 2).

In general, significantly higher AUCs were obtained for all study cohorts, indicating the diagnostic significance of NMJ biomarkers in sarcopenia. Altogether, these findings suggest that the circulating NMJ biomarkers can help diagnose sarcopenia of altering severity in healthy controls and COPD.

### 3.5. Association of Biomarker Levels with the Indexes of Sarcopenia

We further assessed the diagnostic potential of NMJ biomarkers in sarcopenia by investigating their associations with sarcopenia indexes. Multiple regression analysis showed a significant association between the alterations in biomarker panel and HGS during PR (Table 3).

We found significant correlations of ASM, phase angle, and walking speed with the changes in biomarker pane during PR. Among the individual biomarkers, CAF22 and GDNF showed a significant relationship with HGS, while the association of BDNF with HGS failed to reach statistical significance. The ASM, phase angle, and walking speed showed less robust associations with the biomarker panel and individual biomarkers (Table 3). We next evaluated the correlation coefficients of the NMJ biomarkers with sarcopenia indexes and phase angle. HGS showed a significant correlation with CAF22 and BDNF, but not the GDNF in all groups (Table 4). ASMI, phase angle, and walking speed showed varying degrees of correlations with circulating biomarkers. In general, combining the biomarkers into a panel strengthened the degrees of correlation with the HGS, ASMI, and walking speed (Table 4).

## 4. Discussion

We aimed to investigate the dynamic evaluation of sarcopenia in COPD during PR. Our primary finding was the longitudinal correlation of circulating CAF22, BDNF, and GDNF with sarcopenia indexes during the disease course of COPD. Specifically, we found an elevation of CAF22 and reduced BDNF and GDNF plasma levels when diagnosing COPD. PR incompletely restored these biomarkers to normal levels and restored muscle integrity and reduced the incidence of sarcopenia. We also showed that a cumulative risk score representing all three biomarkers enhanced the diagnostic accuracy of sarcopenia. Additionally, SPPB and SARC-F emerged as useful assessment tools of sarcopenia in the elderly.

The plasticity of the NMJ is well-established in various conditions. For example, aging and several diseases result in NMJ deterioration [26,27], while exercise and dietary restriction rejuvenate the NMJ in catabolic states [14]. The active molecular communication among motor neurons, muscle fibers, and PSCs play a pivotal role in repairing NMJs [14]. Plasma CAF22, BDNF, and GDNF are released by all three cellular components of NMJ and may accurately mirror NMJ changes. The elevated level of CAF22 in COPD at diagnosis is consistent with our previous findings [5,13]. Importantly, CAF22 levels show a dynamic association with muscle integrity, mirroring the NMJ plasticity in COPD [5]. The dynamic association between CAF22 levels and sarcopenia is further elicited by an exercise-induced reduction in plasma CAF22 levels along with an improvement in the lean body mass in the elderly [28]. These alterations in CAF22 levels reflect continuous muscle denervation and re-innervation during sarcopenia and PR in COPD. Muscle wasting in ageing is partly due to retraction and death of motor neurons, which results in orphaned fibers. Unless re-innervated by the neighboring neurons, these muscle fibers degenerate and contribute to muscle atrophy. Agrin plays an active role in re-innervation by promoting the recycling of AChRs at the motor endplate. As a consequence, exercise is associated with an upregulation of agrin at the NMJs [29]. Conversely, overexpression of neurotrypsin results in cleavage of agrin and reduced muscle strength and mass [30]. The reduction in plasma CAF22 levels with PR is in line with these findings and indicates an exercise-induced reduction in neurotrypsin levels and consequently a reduced cleavage of agrin in COPD.

We observed downregulation of plasma BDNF and GDNF levels in COPD. Age-related impairment in plasma BDNF and GDNF levels has previously been reported. Growing evidence suggests an association between the regenerative capacity of motor axons and plasma BDNF and GDNF levels [14]. Measurement of BDNF and GDNF together has been a better indicator of axonal regeneration than either of the NFs alone [31]. Following denervation, these NFs are upregulated and assist in NMJ repair and axonal sprouting [27]. The reduced NF levels in COPD indicate the diminished up-regulatory ability of NFs to repair NMJ, which can potentially contribute to the sarcopenia phenotype in COPD patients. The increase in plasma NFs during PR mirrors the effects of exercise in the elderly [14,32] and indicates the protective effects of PR and exercise in COPD.

The combined use of multiple biomarkers proved helpful in risk stratification and diagnosis of sarcopenia, which is supported by higher AUC for the biomarkers panel than the individual biomarkers. The cellular origins of the biomarkers represent the cell types primarily contributing to NMJ degradation [14,27]. Thus, the biomarkers panel can potentially represent multiple pathomechanisms of NMJ instability.

We also demonstrated that simple evaluations such as SPPB and SARC-F show an adequate consistency with sarcopenia phenotype. The components of these tests are associated with poor muscle quality and can be an early assessment tool for sarcopenia. We found that the alterations in SPPB and SARC-F scores during PR were parallel with the restoration of biomarkers levels and sarcopenia indexes, demonstrating the usefulness of these tools for the rapid assessment of sarcopenia. SARC-F is a simple, self-reporting symptom score, while SPPB is a composite test and considers gait speed, balance, and chair stand test for assessment. These tests can be performed in most clinical settings and are recommended for early evaluation of sarcopenia in the high-risk elderly [6,8]. This evaluation can be followed by the measurements of circulating NMJ biomarkers, which require few hours and <1 mL of blood. The blood can be obtained from the sample collected for clinical assays without additional blood sampling.

This study has certain limitations. Sarcopenia has multiple pathomechanisms, and the measurements of additional biomarkers related to other mechanisms of sarcopenia may be required. CAF22, BDNF, and GDNF are also secreted by the non-muscle tissues, including kidneys and the central nervous system. Thus, sarcopenic patients with renal failure and/or neurodegeneration may have aberrant circulating CAF22 and NFs levels, which may not accurately reflect the sarcopenia phenotype. The age range of 61–77 does not represent other age groups. Finally, we did not measure the quadriceps muscle strength, which is an essential predictor of sarcopenia and functional independence [33].

Taken together, we show that despite limitations, the NMJ biomarkers panel may help evaluate sarcopenia and quality of life. Furthermore, the measurement of NMJ biomarkers may follow the evaluation through SARC-F and SPPB and can collectively be adequate assessment tools in predicting and/or diagnosing sarcopenia in the elderly and high-risk groups.

## Figures and Tables

**Table 1:** Body composition and physical parameters of healthy controls and patients with COPD at diagnosis (Dx) and follow-up. Values are expressed as mean ± SD, one-way analysis of variance. * *p* < 0.05 vs. healthy controls; # *p* < 0.05 vs. COPD patients at diagnosis (Dx) (*n* = 77–84 per group).

	Healthy	COPD—At Dx	COPD—Follow-Up
Age at baseline (years)	67.9 ± 5.5	69.3 ± 6.2	70.3 ± 6.3
Body composition			
BMI (kg/m^2^)	25.7 ± 3.5	25.1 ± 3.9	24.9 ± 3.4
ASM (kg)	22.7 ± 3.3	21 ± 2.2 *	21.6 ± 2.6
ASMI (kg/m^2^)	8.3 ± 1.2	7.1 ± 1.6 *	7.5 ± 1.5 *
Percent fat	38.5 ± 4.1	39.2 ± 4.7	36.2 ± 4.9
Phase angle	5.7 ± 0.6	5.31 ± 0.36 *	5.53 ± 0.44 #
Physical Parameters			
HGS (kg)	27.3 ± 5.9	21.4 ± 4.8 *	23.7 ± 4.5 *,#
walking Speed (m/s)	1.28 ± 0.28	1.02 ± 0.21 *	1.16 ± 0.29 *,#
Daily steps count	5375 ± 1137	3136 ± 782 *	6294 ± 893 *,#
Plasma biomarkers			
8-isoprostanes (pg/mL)	48.3 ± 11.23	85.4 ± 19.49 *	62.38 ± 15.38 #
CRP (mg/dL)	0.217 ± 0.027	0.298 ± 0.047 *	0.262 ± 0.039 *,#
Creatine kinase (IU/L)	176.32 ± 35.31	294.3 ± 41.71 *	227.5 ± 49.29 *,#

**Table 2:** The area under the curve (AUC) and confidence intervals for individual biomarkers and the biomarkers panel in healthy controls and patients with COPD at diagnosis (Dx) and follow-up (*n* = 77–84/group).

		AUC	95% C–I	*p* Value
Biomarkers panel	Healthy controls	0.805	0.717–0.839	0.003
	COPD—At Dx.	0.819	0.728–0.874	<0.001
	COPD—Follow-up	0.784	0.694–0.859	0.001
CAF22	Healthy controls	0.739	0.626–0.791	0.026
	COPD—At Dx.	0.778	0.693–0.803	<0.001
	COPD—Follow-up	0.788	0.715–0.831	<0.001
BDNF	Healthy controls	0.731	0.637–0.783	0.001
	COPD—At Dx.	0.769	0.663–0.839	<0.001
	COPD—Follow-up	0.746	0.724–0.862	<0.001
GDNF	Healthy controls	0.783	0.654–0.801	<0.001
	COPD—At Dx.	0.762	0.651–0.794	<0.001
	COPD—Follow-up	0.803	0.704–0.852	<0.001

**Table 3:** Multiple regression analysis investigating the associations of changes in biomarkers levels with changes in handgrip strength (HGS), appendicular skeletal mass (ASM), phase angle, and walking speed in patients with COPD during pulmonary rehabilitation (*n* = 77).

	Coefficient	*p*
Changes in the Biomarker Panel (Log Values) vs. Change in		
HGS	0.272	0.011
ASM	0.103	0.099
Phase angle	0.153	0.066
Walking speed	0.182	0.144
Changes in CAF22 vs. change in		
HGS	–0.316	0.007
ASM	–0.147	0.071
Phase angle	–0.102	0.084
Walking speed	–0.081	0.184
Changes in BDNF vs. change in		
HGS	0.182	0.057
ASM	0.041	0.081
Phase angle	0.148	0.092
Walking speed	0.052	0.121
Changes in GDNF vs. change in		
HGS	0.252	0.021
ASM	0.095	0.144
Phase angle	0.053	0.187
Walking speed	0.092	0.126

**Table 4:** Correlation coefficients of circulating biomarkers with the indexes of sarcopenia and the phase angle in healthy controls and patients with COPD at diagnosis (Dx) and follow-up (*n* = 77–84/group); * *p* < 0.05.

	CAF22	BDNF	GDNF	Biomarkers Panel
HGS				
Healthy controls	0.298 *	0.169 *	0.141	0.194 *
COPD—At Dx.	0.315 *	0.248 *	0.108	0.205 *
COPD—Follow-up	0.341 *	0.271 *	0.129	0.228 *
ASMI				
Healthy controls	0.104	0.118	0.94	0.121 *
COPD—At Dx.	0.094	0.131 *	0.146 *	0.139 *
COPD—Follow-up	0.116	0.084	0.223 *	0.148 *
Phase angle				
Healthy controls	0.103	0.075	0.102	0.095 *
COPD—At Dx.	0.128 *	0.081	0.083	0.089
COPD—Follow-up	0.081	0.059	0.120 *	0.078
Walking speed				
Healthy controls	0.068	0.99	0.147	0.104 *
COPD—At Dx.	0.091	0.113	0.163 *	0.134 *
COPD—Follow-up	0.103	0.106	0.193 *	0.146 *

## Data Availability

Data is available from the corresponding author on request.
